# Ketamine and Calcium Signaling—A Crosstalk for Neuronal Physiology and Pathology

**DOI:** 10.3390/ijms21218410

**Published:** 2020-11-09

**Authors:** Malwina Lisek, Ludmila Zylinska, Tomasz Boczek

**Affiliations:** Department of Molecular Neurochemistry, Faculty of Health Sciences, Medical University of Lodz, 92215 Lodz, Poland; malwina.lisek@umed.lodz.pl (M.L.); ludmila.zylinska@umed.lodz.pl (L.Z.)

**Keywords:** ketamine, calcium, signal transduction, NMDA receptor, AMPA receptor, mTOR signaling, neuronal function

## Abstract

Ketamine is a non-competitive antagonist of NMDA (N-methyl-D-aspartate) receptor, which has been in clinical practice for over a half century. Despite recent data suggesting its harmful side effects, such as neuronal loss, synapse dysfunction or disturbed neural network formation, the drug is still applied in veterinary medicine and specialist anesthesia. Several lines of evidence indicate that structural and functional abnormalities in the nervous system caused by ketamine are crosslinked with the imbalanced activity of multiple Ca^2+^-regulated signaling pathways. Due to its ubiquitous nature, Ca^2+^ is also frequently located in the center of ketamine action, although the precise mechanisms underlying drug’s negative or therapeutic properties remain mysterious for the large part. This review seeks to delineate the relationship between ketamine-triggered imbalance in Ca^2+^ homeostasis and functional consequences for downstream processes regulating key aspects of neuronal function.

## 1. Introduction

**Figure 1:**
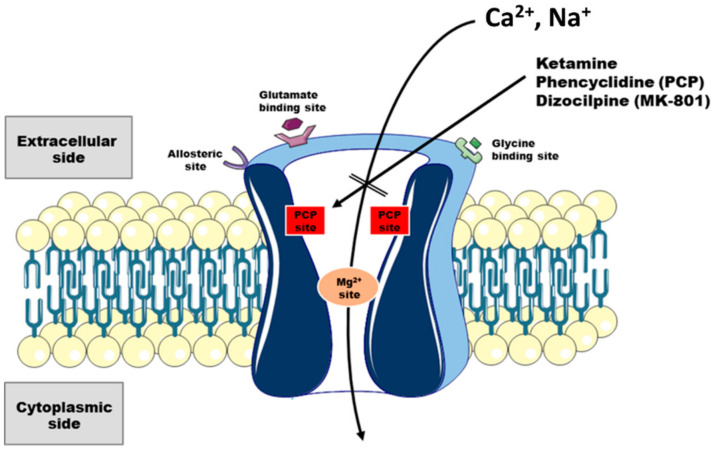
Schematic model of ketamine interaction with NMDA receptor. NMDA receptors are glutamatergic, ligand-gated ion channels comprising of four subunits forming a central pore that is permeable for Ca^2+^. Up to date, seven different subunits have been identified: GluN1, GluN2A, GluN2B, GluN2C, GluN2D, GluN3A and GluN3B. However, NMDA receptor is typically composed of two GluN1 and two GluN2 subunits or a mixture of GluN2/GluN3 subunits. At resting state, the pore is occupied by Mg^2+^, but upon depolarization, the magnesium block is removed allowing Ca^2+^ to enter the cell. Besides depolarization, the activation of the receptor requires concurrent binding of L-glutamate and glycine/D-serine. Pore opening makes it accessible to ketamine which binds to PCP site and blocks further Ca^2+^ influx. The (S)-enantiomer of ketamine binds to the same site. In addition to ketamine and glutamate/glycine binding sites, several other known sites for regulation have been identified in NMDA receptor and they are reviewed in [2,3,5].

Ketamine is a well-established anesthetic drug that has been in use for more than 50 years. Studies in the 1980s demonstrated that ketamine is a non-competitive antagonist of N-methyl-D-aspartate receptor (NMDAR) [1], and the blockage of this receptor appears to largely contribute to anesthetic, analgesic and psychotomimetic, as well as antidepressant effects of the drug [2,3]. Its mechanism of action on NMDAR (Figure 1) is strictly associated with the channel conformational state, as ketamine binds the receptor only when it is open producing, like its structural analogs—phencyclidine (PCP) and dizocilpine (MK-801), a trapping type of open channel block. The immediate effect is the occlusion of Ca^2+^ and Na^+^ flow through the channel pore and pronounced reduction in both, frequency and the mean open time of NMDA receptor, therefore, preventing neuronal activation required for conscious state [2,4]. Due to its slow-off rate, ketamine remains trapped even after channel closing, which accounts for its long-lived block that is relieved by upcoming event of opening. Membrane depolarization, for instance, is known to reduce this block presumably by speeding up drug dissociation, although the underlying mechanism of voltage-dependence has not been completely resolved [3,5]. Due to its high trapping potency, ketamine causes a prolonged tonic blockade which disrupts neuronal physiology. Indeed, the systemic administration of ketamine in humans provoked both acute and sustained effects. Acutely, subjects within 1 h of administration experienced a wide spectrum of psychotomimetic and cognitive aberrations mimicking the ones observed in schizophrenia [6]. On a larger timescale, many of the clinically beneficial effects were observed way after the drug has been completely excreted from the body. The best example is currently the widely investigated antidepressant effect of ketamine, which remains for about a week [7], despite short half-life of ketamine in humans lasting 2–4 h when administered intravenously [2]. The dual, concentrated and time-dependent effects are thought to arise from low ketamine selectivity toward NMDAR and the drug interference with multitude signaling pathways. The ongoing studies are perpetually identifying new molecular targets for the drug (reviewed in [3,5,8,9]), and currently, the contribution of GABA(γ-aminobutyric acid)ergic, dopaminergic, serotonergic, cholinergic, aminergic and opioid systems to positive and negative modulatory role of ketamine has been confirmed. These studies also disclosed, broader than was previously considered, the clinical relevance of ketamine metabolites and the differential potency of ketamine’s racemic forms. Nonetheless, even if the behavioral changes elicited by ketamine are well-established and have superb translational potential, the mechanism(s) by which the drug may exert its multifaceted action is highly complex and involves a crosstalk among numerous signaling pathways. In neurons, the unifying second messenger that is either directly or indirectly engaged in virtually all systems of signal transduction is calcium. Moreover, the growing body of evidence suggests that ketamine-induced, NMDAR blockage-dependent impairments in neuronal calcium homeostasis may be pivotal for understanding why ketamine produces such a wide spectrum of behavioral abnormalities. In this review, we focus on the interaction of ketamine with neuronal calcium toolkit and discuss the potential consequences of drug-induced Ca^2+^ imbalance on neuronal development and synaptic transmission. 

## 2. Ketamine and Neuronal Calcium Homeostasis

### 2.1. Ca^2+^ Homeostasis in Healthy Neurons

**Figure 2:**
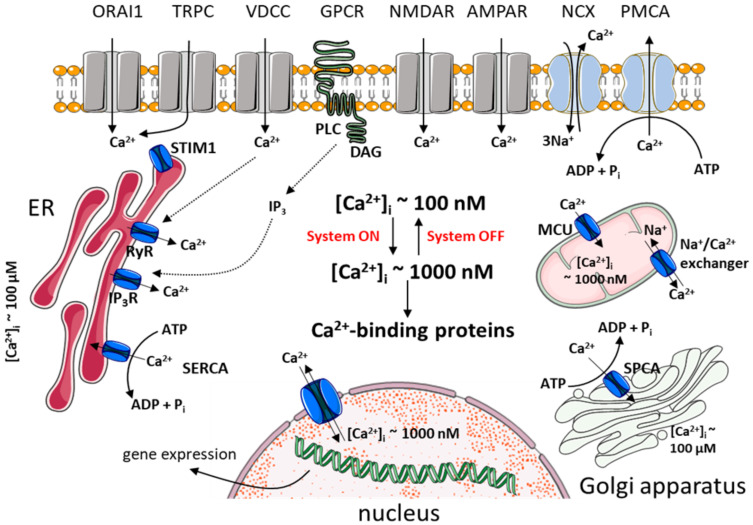
Calcium homeostasis and signaling in healthy neurons. Ca^2+^ can enter the cell during the “on” phase through voltage-dependent calcium channels (VDCCs), transient receptor potential channels (TRPCs), calcium release-activated calcium channel (ORAI1) or through glutamate-activated receptor-operated channels: N-methyl-D-aspartate receptors (NMDARs) and α-amino-3-hydroxy-5-methylisoxazole-4-propionate acid receptors (AMPARs). Calcium can be mobilized from the ER upon activation of some G protein-coupled receptors (GPCRs) via inositol-1,4,5-triphosphate (IP_3_) receptors (IP_3_Rs) or its release may occur through ryanodine receptors (RyRs). Depletion of the ER is followed by the activation of store-operated calcium entry (SOCE) which is based on the interaction between ORAI1 and the stromal interaction molecule 1 (STIM1). Intracellular Ca^2+^ increases are buffered by calcium-binding proteins but some Ca^2+^ signals can reach the nucleus and affect gene transcription. During the “off” phase, cytosolic Ca^2+^ is sequestered by sarco(endo)plasmic reticulum Ca^2+^-ATPase (SERCA), secretory pathway Ca^2+^-ATPase (SPCA) or mitochondrial uniporter (MCU) to the ER, Golgi apparatus and mitochondria, respectively, or transported to the extracellular milieu by plasma membrane Ca^2+^-ATPase (PMCA) and Na^+^/Ca^2+^ exchanger (NCX). Ca^2+^ in the nucleus is controlled independently.

The control of intracellular Ca^2+^ concertation [Ca^2+^]_i_ is of paramount importance for neurons, as it determines their survival and physiological utility since early neurogenesis through function as mature cells. In neurons, Ca^2+^ plays a dual role as a charge carrier and ubiquitous second messenger. This dual nature allows for the generation of a broad spectrum of signals, which can be distinguished by spatial and temporal dimensions, amplitude, frequency of oscillations or localization to discrete neuronal compartments [10,11,12,13]. The readout of these signals by local or global Ca^2+^-sensing proteins and subsequent transmission of decoded message to cellular effectors underlies the involvement of calcium in a wide range of crucial generic process, such as apoptosis, gene transcription or proliferation. In neurons, concerted action of cellular systems allowing for Ca^2+^ influx and those responsible for Ca^2+^ extrusion allows to generate unique pattern of transient (microseconds to minutes) increases in cytoplasmic Ca^2+^ concertation, ranging from approximately 100 nM at rest to 1–500 µM after electrical- or receptor-mediated stimulation. This ability to control intracellular Ca^2+^ rises is peculiar to both developing and mature neurons. Calcium-mediated signaling controls specification of neuronal subtype and the regulation of dendritic growth, arborization, axonal outgrowth and subsequent organization into neurotransmitter subtype. The proper outcome of developmental neurogenesis further determines the mature synaptic transmission, which primarily depends on the coupling between membrane depolarization and intracellular Ca^2+^ increases. Because neuronal life depends, perhaps to a greater degree than other eukaryotic cells, on precise orchestration of these Ca^2+^-regulated processes, neurons have been equipped with complex homeostatic mechanisms that function together to initiate and propagate Ca^2+^ signal and yet to counterbalance potentially detrimental effects of abnormal rises in [Ca^2+^]_i_. The overview of Ca^2+^ homeostasis toolkit in healthy neurons is depicted in Figure 2. 

#### 2.1.1. Mechanisms that Turn on Ca^2+^ Signal

An increase in [Ca^2+^]_i_ can reflect the influx from extracellular milieu through two types of membrane Ca^2+^ channels: voltage-dependent Ca^2+^ channels (VDCCs) and ligand-gated ionotropic channels (LGIC) or the release from intracellular reservoirs, mainly endoplasmic reticulum (ER), where Ca^2+^ concentration is nearly 1000 higher than in cytosol. 

The main types of LGICs activated by excitatory neurotransmitter glutamate are NMDAR, α-amino-3-hydroxy-5-methyl-4-isoxazolepropionic acid receptors (AMPAR) and kainate receptors. An effective route of calcium entry into neurons through NMDARs has been implicated in neuronal differentiation, migration, synaptogenesis, synaptic remodeling and long-lasting synaptic processes such as long-term potentiation (LTP) and long-term depression (LTD) [14]. 

Dysfunctional NMDARs have been repeatedly reported in a number of brain disorders [14]. NMDAR are heteromeric assemblies of NR1, NR2 and NR3 subunits, which form functional channels that differ by physiological and pharmacological properties [15]. Assembled receptors are targeted to the postsynaptic densities (PSD) and frequently localize, together with AMPARs at nascent synapses [16]. PSDs are the sites where NMDARs and AMPARs, through the members of the postsynaptic density protein-95 (PSD-95) family of scaffolding proteins, are structurally and spatially organized into large multimolecular signaling complexes [17]. The composition of such complexes may vary according to the stage of development or functionality of particular brain regions but in overall, it provides an additional level of response specificity. The NMDAR-mediated rise in postsynaptic Ca^2+^ may activate a network of kinases and phosphatases, and other calcium-sensitive downstream targets that promote persistent changes in synaptic strength. 

Voltage-dependent calcium channels (VDCCs), found in the plasma membrane of all excitable cells, are key transducers of cell surface membrane potential changes into intracellular calcium transients that initiate different physiological processes in neurons. So far, multiple distinct representatives of VDCCs have been identified, including L-type sensitive to dihydropyridines, the N-type and P/Q-type responsive to conotoxins and T-type responsive to ethosuximide. High diversity among these channels, their peculiar location and distinct biophysical properties are thought to confer a specific neurophysiological response. For instance, L-type channels are mainly found in cell bodies and dendrites whereas high-threshold VDCCs such as N- and P/Q-type are primarily located in the axonal boutons and are involved in neurotransmitter release [18]. 

Ca^2+^ release from the ER in neurons is facilitated by ryanodine receptors (RyRs) and inositol-1,4,5-triphosphate receptors (IP_3_Rs). Agonist-mediated stimulation of G-protein-coupled receptors (GPCRs) or tyrosine kinase-linked receptors triggers a cascade that leads to the phospholipase C (PLC)-mediated generation of diacylglycerol (DAG) and inositol 1,4,5-triphosphat (IP_3_). DAG can directly activate several targets including transient receptor potential canonical channels (TRPCs) whereas IP_3_ activates its cognate receptor leading to ER Ca^2+^ store depletion. Sudden rise in [Ca^2+^]_i_ is a major trigger for further Ca^2+^ release through RyRs in a process called Ca^2+^-induced Ca^2+^ release (CICR). The CICR is of paramount importance for shaping Ca^2+^ signal as it couples the coordinative opening of channel(s) observed as calcium “sparks” or “puffs” to the generation of widespread calcium waves or oscillations [19,20]. Depletion of the ER activates a refilling mechanism knows as capacitative calcium entry (CCE). The Ca^2+^ sensor STIM1 senses the changes in Ca^2+^ concentration in the ER and subsequently gates Orai1, which functions as Ca^2+^ channel in the plasma membrane, as well as TRPC channels, which tend to interact with IP_3_Rs. Indeed, Ca^2+^ entry through TRPC channels has been demonstrated to play fundamental role in basic fibroblast growth factor (bFGF)-induced proliferation of neural stem cells [21]. In this process, activation of TRPC1, but not TRPC3, had a dominant effect. Contrary, both TRPC1 and TRPC3 controlled the switch between temperature-sensitive proliferation and differentiation of SV40 T antigen-immortalized precursors of hippocampal neurons (H19-7) [22]. TRPC3 and TRPC6, but not TRPC1, have been shown to be associated with brain-derived neurotrophic factor (BDNF)-dependent neuronal outgrowth [23]. Moreover, TRPC-mediated ionic conductance was shown to be able to depolarize neuronal membrane, presumably activating VDCCs [24]. 

#### 2.1.2. Mechanisms that Turn off Ca^2+^ Signal

Neuronal calcium clearance mechanisms control the amplitude and the duration of intracellular Ca^2+^ transients being primarily responsible for the restoration of [Ca^2+^]_i_ to its basal level following stimulation [20]. This is achieved through rapid buffering by cytoplasmic Ca^2+^-binding proteins, mainly calmodulin, calcineurin, calretinin, parvalbumin or calbindin, and coordinated action of sophisticated toolkit allowing for sequestration or removal of Ca^2+^ excess. The latest is facilitated by calcium pumps and exchangers. Calcium ions are pumped out against the concentration gradient by plasma membrane-located Ca^2+^-ATPases (PMCA1-PMCA4) and Na^+^/Ca^2+^ exchangers (NCX1-3). The internal structures, such as ER, mitochondria and Golgi apparatus possess their own release and refilling mechanisms, which not only contribute to Ca^2+^ clearance during the “off” phase, but also to shaping intracellular Ca^2+^ signaling. The sarco(endo)plasmatic reticulum Ca^2+^-ATPase (SERCA) represented in neurons by two isoforms: SERCA2 and SERCA3, decreases [Ca^2+^]_i_ by pumping Ca^2+^ to the cisterns of the ER where it is further sequestered by luminal EF-hand proteins [19,20]. Besides, the well-know SERCA pump, secretory pathway Ca^2+^-ATPase (SPCA), which has functioning that is much less characterized, transports both Ca^2+^ and Mn^2+^ into the Golgi lumen and is involved in the cytosolic and intra-Golgi homeostasis of these ions. Mitochondria, especially those in close proximity to the ER, can accumulate significant amount of Ca^2+^ through mitochondrial uniporter (MCU) while the Na^+^/Ca^2+^ exchanger is proposed to be the primary mechanism for mitochondrial Ca^2+^ extrusion in excitable cells [25]. By sequestering and releasing Ca^2+^, mitochondria serve as important regulators of cellular metabolism, ATP production and cell death. 

### 2.2. Interaction of Ketamine with Neuronal Ca^2+^ Toolkit

The association of ketamine action with potential modifications of Ca^2+^ homeostasis is now brought back to the forefront of ideas of molecular etiology of drug-induced psychiatric effects. Indeed, growing body of evidence suggests that one of the critical neuronal function targeted by ketamine is Ca^2+^-dependent signaling [26,27]. This feature of ketamine, which can be partially deduced from animal models, arises from a reduction of NMDAR drive and subsequent down regulation of Ca^2+^-sequestering proteins as discussed by Lidow [26]. Inhibition of NMDARs located on GABAergic interneurons attenuates GABA release leading to overactivation of major excitatory pathways. According to the hypothesis of disinhibition, blockage of NMDAR by ketamine should induce massive Ca^2+^ entry through glutamate-independent pathways and mobilization from intracellular stores, thus producing prolonged calcium dynamics and increased [Ca^2+^]_i_ in large population of neurons. To support it, sustained elevations in basal cytosolic Ca^2+^ concentration were seen in functionally different regions of rat brain following in vivo ketamine administration (30 mg/kg). Interestingly, a positive correlation between ketamine-induced hyperlocomotion and [Ca^2+^]_i_ in the cortex and cerebellum was demonstrated [28]. In primary rat hippocampal neurons isolated at E19, application of ketamine (3 µM) resulted in an increase in cytosolic Ca^2+^ concentration, an effect that was observed 24 h after drug washout [29]. Further increase in ketamine concentration (10 µM or higher) produced significant and dose-dependent rise in [Ca^2+^]_i_ seen already 15 min after washout. In another study, Ali and coworkers, using two-photon calcium imaging, found that ketamine suppressed the activity of somatostatin-expressing interneurons, which are a fraction of GABAergic interneurons, in the medial prefrontal cortex but not in primary motor cortex, suggesting regionally specific effect [30]. They further showed that ketamine disinhibited apical dendrites, leading to elevated Ca^2+^ transients in the apical dendritic spines of pyramidal neurons within the first hour of systemic administration. 

So far, only few studies have been attempted to resolve the mechanism(s) underlying [Ca^2+^]_i_ rise induced by ketamine. According to one hypothesis, inability to maintain brain Ca^2+^ homeostasis may be a result of imbalanced expression between calcium “off” and “on” systems. An extended analysis of the expression of genes encoding main players in neuronal Ca^2+^ handling showed that ketamine predominantly affected the expression of VDCCs and membrane transporters responsible for Ca^2+^ extrusion (Table 1). However, no uniform pattern can be seen between analyzed brain regions what may account for their functional specificity and different susceptibility to ketamine [28]. The key changes correlating with increased [Ca^2+^]_i_ involved ATP-dependent Ca^2+^ pumps, in particular PMCA2 and PMCA3 [28]. Despite some potential adaptations in the form of increased expression of constitutive isoforms PMCA1 and PMCA4 or SERCA pumps reported in the same study, the neuronal calcium imbalance upon ketamine treatment seems to be persistent and irreversible. This is supported by a large number of studies showing the importance of PMCA and SERCA pumps in Ca^2+^ homeostasis maintenance in a variety of neuronal models [19,20,31,32,33].

Further, studies on rats challenged with 30 mg/kg ketamine demonstrated that basal hydrolytic and transferase activities of PMCA, as well as stimulatory effect of calmodulin were significantly reduced in synaptosomal membranes isolated from cortex, cerebellum, hippocampus and striatum. Moreover, immunoprecipitation of ^32^P-labeled PMCA-ATP complexes showed no preference of ketamine toward any of the PMCA isoforms [34,35]. In vitro experiments with purified PMCA digested with trypsin, which is expected to remove C-terminal regulatory domain, demonstrated that this enzyme is a direct target for ketamine [35]. Both intact and truncated versions of PMCA were, although unequally, inhibited by the drug suggesting the existence of two independent ketamine-PMCA interacting sites. Further competitive experiments disclosed that one of the site can be located within calmodulin-binding domain and the other, in a large catalytic loop containing the site of phosphointermediate formation. Besides affecting the activity of PMCA, ketamine administration influenced the amount of PMCA isoforms in synaptosomal membranes in a manner dependent on brain region. Significant decrease of PMCA3 immunoreactivity was prominent in cerebellum, hippocampus and striatum while the modest decrease in PMCA1 level was only in the hippocampus. Compensatory up-regulation of PMCA4 and PMC1 was in turn demonstrated in cortex and cerebellum, respectively [34]. Although no specific studies were dedicated to elucidating functional consequences of ketamine’s effect on PMCA, such deep modifications are expected to affect neuronal potency for Ca^2+^ extrusion and the activity of PMCA-downstream signaling cascades.

It is known that location and function of PMCA at the pre- and postsynaptic sites is regulated by interaction with postsynaptic-density-95/discs large/zona occludens-1 domain (PDZ-domain)-containing proteins, mainly PSD95 scaffold. The interaction between PSD95 and PMCA is thought to tether the pump to a specialized high calcium membrane microdomains also rich in NMDA receptors. This raises the possibility of multiprotein complex formation coupling NMDAR-mediated Ca^2+^ influx to the pumping activity of PMCA. Interestingly, an enhanced formation of NMDAR1/PSD95/PMCA4 complexes was demonstrated in cortical preparations isolated from ketamine-treated animals (30 mg/kg) what could be seen as a rescue for reduced NMDAR-driven downstream signaling and a counterbalance for higher Ca^2+^ influx through receptors other than NMDAR. The potential existence of PMCA, PSD95 and NMDAR within a ternary complex was also considered as one of the mediators of enhanced glutamate release from cortical and striatal synaptosomes isolated from ketamine-treated animals. Calcium-dependent glutamate release has been found to control many aspects of neuronal development and synaptogenesis [36]. In line with this, ketamine administration in utero was consistently linked with inhibition of neurite outgrowth and disturbance in maturation of pyramidal neurons in prefrontal cortex of offspring [37], which may affect the behavior later in adulthood. 

## 3. Ketamine, Calcium and Neuronal Development

### 3.1. Ketamine and Calcium Oscillations

From a historical perspective, neuronal development was largely attributed to the changes in gene expression. It has recently been apparent that during differentiation of majority of neurons, these changes are governed by electrical activity acting through downstream effectors [38,39,40,41,42]. During development, Ca^2+^ is the most ubiquitous second messenger which role is closely associated with the progenitor cell fate specification, migration, differentiation, axonal outgrowth, dendritic arborization, synaptogenesis in the brain and the spinal cord and neuronal apoptosis [42,43,44]. The spontaneous Ca^2+^ transients correlating temporary and spatially to neural induction, have been observed as early as gastrulation and later linked to the expansion of neural progenitor pool [43]. As shown for rodents, these oscillations exhibit a recurrent and transient developmental pattern, which can be seen at postnatal day 1 and ends up at around day 14 [45]. It has been grounded in the literature that the somatic and axonal Ca^2+^ transients accompany spiking activity whereas dendritic and spinal elevations are associated with synaptic inputs. Besides this classical view, the newest data suggest that cytosolic Ca^2+^ transients are much more complex in nature, occur synchronously with mitochondrial Ca^2+^ oscillations [46] and their compartmentalization depends on the type of neuron and its developmental stage [47]. 

In the newest excellent experiments, synaptic Ca^2+^ transients and deficient dendritic inhibitory mechanism produced by subanesthetic ketamine (10 mg/kg) were demonstrated to be important alterations for a local cortico-cortical connectivity [30] and the growth of dendritic spines during neuronal development [48]. Using postnatal day 1 immature hippocampal neurons, Huang and colleagues demonstrated that the frequency and amplitude of Ca^2+^ oscillations in vitro was 0.042 ± 0.006 Hz, and 2.01 ± 0.07 Hz, respectively, a value that was noticeably increased in the presence of 1–10 μM ketamine [49]. Interestingly, administration of ketamine at a concentration similar to that achieved in the cerebrospinal fluid (100–200 µM) [50] significantly reduced the amplitude of Ca^2+^ peaks in these neurons without affecting the frequency and the number of oscillations. The administration of ketamine at concentrations that are higher than the clinical concentration of 300 μM abolished oscillatory activity in a dose-dependent manner. As NMDAR is known to contribute to intracellular Ca^2+^ transients [51], this substantial inhibitory effect may reflect gradual decrease in NMDAR channel frequency and opening time in the presence of increasing ketamine concentration. In the same paper, it was suggested that ketamine-dependent inhibition of Ca^2+^ transients in immature neurons may be co-mediated by GABA_A_ receptor. In contrast to the adult brain, in the developing hippocampus, GABA acts as excitatory neurotransmitter and depolarizes developing pyramidal neurons through GABA_A_ receptor [52]. At this stage of development, GABA_A_ receptor inhibition should lead to suppression of spontaneous Ca^2+^ transients. However, several studies demonstrated increase in Ca^2+^ periodical oscillations in the presence of GABA_A_ receptor antagonist bicuculline [53,54]. This is in line with the recent findings showing increased activity of high affinity GABA_A_ receptors in hippocampal and cortical neurons [55], and enhancement of GABA current and synaptic function [56] by clinically-relevant concentrations of ketamine. The complete suppression of cortical activity in neonatal animals was only achieved by combined administration of ketamine (40 mg/kg) and the positive GABA_A_ receptor allosteric modulator midazolam (9 mg/kg) [57]. The crosstalk between NMDAR and GABA_A_ receptor in developing neurons is further supported by induction of Ca^2+^ oscillations in cortical neurons in the absence of Mg^2+^. These findings can be described as being consistent with recently proposed role of GABA_A_-mediated inhibitory postsynaptic potentials and individual Ca^2+^ spikes in the regulation of excitability and plasticity during development of pyramidal neurons [58,59].

Another study on immature hippocampal neurons isolated from embryonic day 19 (E19) demonstrated stereoselectivity of ketamine toward reversible suppression of the amplitude and frequency of Ca^2+^ oscillations [53]. The S(+) ketamine reduced the frequency more potently than the R(-) enantiomer for concentrations up to 100 µM. The 50% effective concentration for ketamine-induced reduction of Ca^2+^-oscillation frequency was approximately 25 and 150 μM for the S(+) and R(−) ketamine giving a potency ration of 1:6. In case of amplitude, the 50% effective concentration was approximately 20 µM and 60 µM for the S(+) and R(−) ketamine, respectively, giving S(+)/R(−) potency ratio for amplitude attenuation of 3:1. These observations correlated with 3.4-fold larger potency of the S(+), compared with the R(−) ketamine regarding its anesthetic, a 4-fold larger potency regarding its analgesic, and a 4.8-fold larger potency regarding its amnesic effect [60]. Interestingly, when neurons were incubated with S(+) ketamine for longer time period (24 h), the inhibitory effect toward Ca^2+^ oscillation was seen at the concentration as low as 3 µM [29]. Using in vivo two-photon imaging, Yuryev and colleagues confirmed the inhibitory effect of ketamine toward spontaneous calcium transients in the marginal zone of the cortex of E15-E15 embryos connected to the mother through umbilical cord [61]. These transients were selectively sensitive to maternal or in situ application of ketamine but not maternal isoflurane anesthesia. This is in agreement with the fact that functional NMDA receptors are present at the early stages of brain development and they are important for generation of spontaneous calcium activity in neuronal development and maturation. 

It has been demonstrated that S(+) ketamine applied at 3 µM concentration during the phase of intensive synaptogenesis, which normally occurs between days 14 and 21 in culture, severely affected the dendritic arbor development and the number of branching points [29]. This process was associated with the reduction of Ca^2+^/calmodulin-dependent protein kinase II (CaMKII) activity and decreased expression of synapsin at the drug concentrations that were too low to induce apoptosis (3 µM or 10 µM) [29]. It is well-established that Ca^2+^ oscillations in neurons activate CaMKII, which is involved in neuronal development, plasticity and memory formation [62]. CaMKII may in turn phosphorylate synapsin I at Ser-566 and Ser-603, linking neuronal plasticity to Ca^2+^ signaling. However, CaMKII is not highly expressed at early development stages and becomes readily detectable in the entire population of stage V neurons [63]. This observation is consistent with the hypothesis of functional coupling between CaMKII and synapsin I, as the presynaptic pool of CaMKII and synapsin I phosphorylation increase in parallel with synaptogenesis and synaptic maturation. The relationship between calcium, CaMKII, neuronal density and general anesthesia was confirmed in another study showing increase in CaMKII immunoreactivity 24 h after treatment with 5 or 25 mg drug/kg in the hippocampus of 10-day-old mouse pups [64]. The increase in CaMKII was also demonstrated during 5 h ketamine treatment (30 mg/kg), which was accompanied by higher number of dendritic spines but decreased diameter of spine head in somatosensory cortex and hippocampus. Recording of Ca^2+^ signals showed that majority of newly formed spines was responsive and functional, however these phenomenon was observed only in younger animals (P15) and the potency of ketamine to promote spines growth was lost at P30 [65]. Therefore, attenuation of NMDA receptor-induced Ca^2+^ oscillations by ketamine is expected to impair a network architecture in developing brain. 

### 3.2. Ketamine, Calcium and Enhanced Neuronal Death

Apoptosis occurs extensively as a physiological process during brain development and has been reported in both mitotic neuronal precursors and postmitotic differentiated neuronal populations [66]. It is estimated that one-third of these cells is eliminated within first two weeks after birth. While the removal of excessive neurons is necessary to establish functional circuity, disturbance in developmental apoptosis may lead to enhanced neurodegeneration. Cytosolic calcium oscillations that exceed a certain temporal and spatial scope are recognized as apoptotic or necrotic trigger, thus influencing neuronal death [67]. However, the consequences of excessive Ca^2+^ signal may vary according to their duration, magnitude and type of neuron which is primarily affected. 

More than 20 years of in vitro and in vivo research demonstrated that ketamine is able to induce neuronal cell death in immature neurons. These studies identified ketamine-induced neuronal degeneration mostly as apoptotic in nature providing a mechanism for the anesthetic-induced developmental toxicity. Ketamine-induced neuroapoptosis has been demonstrated in seven-day-old mice, in the cortex of 122 days of gestational and five-day postnatal rhesus monkey and in human neurons differentiated from stem cells [36,68]. The recent studies in mice of postnatal ages 5 to 12 (P5–P12) demonstrated that even a single dose of 30 mg/kg ketamine, a dose that provides an adequate surgical plane of anesthesia, could induce apoptosis of cortical neurons, which followed a very similar time course to that of physiological apoptosis. Reduced neuronal activity was shown to affect the pattern of ketamine-induced apoptosis and shift the cortical lamination to a more immature form [69]. Increased apoptotic death induced by ketamine was further showed to coincide with suppression of neuronal oscillations, and persistent and irreversible increase in intracellular Ca^2+^ concentration [29]. 

With regard to in vitro models, important relevant question can be raised: how ketamine produces a permanent loss of intracellular calcium homeostasis ultimately leading to neuronal death? Several reports suggested that neurotoxicity is associated with some indirect actions of ketamine or induction of compensatory mechanisms rather than in situ drug concentration in the blood and the brain [70,71]. It has been reported that the expression of NR1 subunit of NMDA receptor was significantly elevated in rat neonatal brain following ketamine administration [71]. This compensatory up-regulation allows for accumulation of intracellular Ca^2+^ to potentially toxic concentrations what is hypothesized to be responsible, or at least significantly contribute to ketamine-induced neuronal death. Another set of data supporting calcium hypothesis of ketamine neurotoxicity comes from in vitro live-cell neuronal imaging using calcium-sensitive sensors. These studies showed that Ca^2+^ influx induced by activating concentrations of NMDA was significantly higher in primary neuronal cultures exposed to ketamine [72,73]. NMDA-evoked increases in intracellular Ca^2+^ were blocked when neurons were perfused with EGTA-containing buffer suggesting the extracellular rather than endoplasmic reticulum origin of Ca^2+^ rises [72]. It was further proposed that Ca^2+^ overload caused by up-regulated NMDA receptors exceeds mitochondrial buffering capacity and promotes excessive production of ROS by electron transport chain complexes [72,74]. The role of Ca^2+^ as a trigger for ROS generation is well known and has been widely discussed elsewhere [75], similar to the apoptosis signaling pathways activated by oxidative stress [76,77]. Chronic administration of low doses of ketamine (0.5 µM) was shown to increase neuronal superoxide production mediated by NADPH oxidase [78]. This phenomenon was observed in the prefrontal cortex, CA3 region in the hippocampus and the reticular nucleus of the thalamus suggesting that increased activity of NADPH oxidase throughout the brain after drug exposure may be pivotal to the loss of phonotype of fast-spiking interneurons. These observations are consistent with the view that the presence of functional NMDA receptors in neurons and concomitant higher intracellular Ca^2+^ load are critical for ketamine-induced neuronal death. The absence of functional NMDA receptors can then explain why clinically-relevant ketamine concentrations are ineffective to induce apoptosis in undifferentiated neural stem cell [73].

The early study of Ikonomidou et al. [79] revealed that ketamine-induced widespread apoptotic neurodegeneration in neonatal brain was restricted only to neurons. This finding was further confirmed in differentiated neural cells where ketamine affected only polysialic acid cell adhesion molecule (PSA-NCAM)-expressing neurons but not astrocytes or oligodendrocytes [80]. The antisense oligonucleotides preventing both NR1 up-regulation and massive Ca^2+^ influx reversed the ketamine effect on PSA-NCAM expression and protected neurons from apoptosis. The primary role of PSA-NCAM is to regulate neurite outgrowth and target recognition during brain development, where it facilitates neuronal pathfinding and migration of neural projections [81,82]. Several lines of evidence suggest that Ca^2+^ entry through NMDA receptor is important for expression and activity of PSA-NCAM during postnatal development and glutamate receptor-dependent Ca^2+^ mobilization stabilizes the synapses on PSA-NCAM-expressing neurites [83,84]. Moreover, in developmentally synchronized population of PSA-expressing motor neurons, de novo biosynthesis of PSA and its transfer to NCAM molecule were sensitive to the perturbations in Ca^2+^ pool in the intracellular compartments [85]. Therefore, it was proposed that NCAM-dependent neurite outgrowth may be driven by compartmentalized calcium signaling initiated by a variety of Ca^2+^-sensitive membrane channels and receptors with NMDA receptor being an obvious candidate. Further studies demonstrated that antagonists of L- and N-type voltage dependent Ca^2+^ channels (VDCC) can substantially inhibit the response of cerebellar neurons isolated at postnatal day 9 to NCAM and lead to neuronal death. The contribution of VDCCs to developmental neurotoxicity of ketamine was also demonstrated in PC12 cells and primary hippocampal neurons [86,87]. In the latter, treatment with nimodipine, an L-type VDCC blocker, prevented the elevations in intracellular Ca^2+^, leading to depression of apoptotic pathways and restoration of neuronal functions. This protective effect is consistent with the ability of ketamine to inhibit L-type VDCC current [88]. 

### 3.3. Effects on Neurogenesis 

#### 3.3.1. Effects Facilitated by NMDA Receptors

Increasing the number of studies suggests a powerful influence of Ca^2+^ signaling on various aspects of neurogenesis, from proliferation and migration of neural progenitor cells to differentiation [89]. Both spontaneous Ca^2+^ oscillations, as well as activity driven Ca^2+^ influx through voltage-dependent calcium channels, that confer neuronal excitability and outgrowth, are implicated in these events and several key protein players have been identified as direct or indirect targets for ketamine [5]. Although this list is rapidly growing, the mechanisms by which ketamine may affect neurogenesis are still a matter of debate. 

The current findings indicate that endogenous overactivation of NMDA receptors and massive Ca^2+^ influx are expected to interfere with the incorporation of newly born neurons into functional brain circuits. To support that, it has been demonstrated that blockage of NMDA receptor-mediated Ca^2+^ entry by ketamine is linked with higher number of newly generated neurons in the rat hippocampus [90]. This is further in line with the results of NMDA receptor genetic ablation showing increased density of functional synapses in developing CA1 pyramidal neurons [91]. Contrary, Luk and colleagues [92] revealed that in utero exposure to NMDA antagonists dramatically slowed down the rate of neurogenesis in striatum. The regional variations in the proliferation rate in the germinal zones of the telencephalon were suggested to be important factor influencing the neuronal phenotypic diversity in the forebrain [92]. This may, at least in part, explain some discrepancies in the experimental findings, which unanimously demonstrate the complexity of ketamine-evoked changes in the developing brain. 

One possible mechanism by which ketamine may affects proliferation of precursor cells is the attenuation of c-Fos and c-Jun expression and subsequent downstream inhibition of AP1 transcription factor assembly. This effect can be largely ascribed to the inhibition of NMDA receptor, as the blockage of Ca^2+^ influx in dentate gyrus neurons prevented c-Fos mRNA induction in response to NMDA receptor agonists [93]. However, one study demonstrated that ketamine administered at behaviorally active doses (25 and 50 mg/kg) significantly induced the transcripts of immediate-early response genes, including c-Fos, in cortical regions [94]. Even despite this inconsistency it appears that NMDA receptor and concomitant Ca^2+^ influx can regulate the expression of these genes, which are engaged in neural progenitor cell proliferation.

A second line of evidence is provided by the NMDA receptor-Ca^2+^-nitric oxide signaling. Nitric oxide (NO) is a well-established regulator of neurogenesis and its dual role in this process has been described [95]. On one hand, NO increases the proliferation of neural stem cells and its pro-neurogenic role was revealed in the subventricular zone (SVZ), on the other it impairs neurogenesis in the physiological conditions [95]. The calcium-sensitive neuronal isoform of nitric oxide synthase (nNOS) is well known to be enhanced by NMDA receptor-mediated Ca^2+^ influx. The blockage of this receptor by ketamine resulted in reduced NO synthesis and promoted the production of new neurons [90]. Similar effects were seen in the hippocampal dentate gyrus of nNOS-knockdown mice [96]. Contrary to the neuron-derived nNOS, neural stem cell-derived enzyme activity was required for neuronal differentiation. The repression of nNOS significantly decreased the rate of this process while nNOS upregulation promoted generation of new neurons from the progenitor cells [97]. Recent evidence from nNOS^-/-^ mice suggests a pivotal role of Ca^2+^ signaling in NO-dependent regulation of Purkinje neuron (PN) dendritic development [98,99]. According to these studies, overactivation of mGluR1 receptor in parallel and climbing fibers leading to overload of intracellular Ca^2+^ in PN is responsible for structural malformations due to the activation of calcium-dependent proteases. Altered size and time course of Ca^2+^ dynamics within nNOS^-/-^ PN may also contribute to changes in dendritic spine morphology—from mushroom-like shape to thin-type spines. Therefore, blockage of NMDA receptor-dependent Ca^2+^ entry by ketamine is expected to affect intracellular Ca^2+^ transients within PN and suppress NO signaling leading to the inhibition of neurite elongation and growth cone movement. Such dendritic deficits were seen in nNOS^-/-^ cerebellum during early development. However, the extent of the contribution of calcium and NMDA receptor to the regulation of neurogenesis by NO would strongly depend on the subunit composition of the receptor. 

It has been suggested that the effect of ketamine on synapse formation and synaptic activity is mediated through the NMDA receptor containing NR2B subunit [100,101]. NR2B is also a predominant NR2 subunit in human and rat neural stem cells. Therefore, NR2B-containing NMDA receptors are supposed to be critical in the regulation of neurogenesis. The expression of NR2B subunit is also thought to be required for a form of plasticity seen exclusively in younger neurons and therefore NMDA receptor containing this subunit are considered as developmental form of NMDA receptor [102]. The conductance and calcium permeability were nearly identical for NR2A- and NR2B-containg NMDA receptor, although experimental findings suggest that post-translational modifications of synaptic NR2B-containg receptor may enhance fractional Ca^2+^ current and let more Ca^2+^ to enter. This effect is putatively responsible for the greater mitochondrial Ca^2+^ accumulation upon activation of these receptors, what may indicate the mechanism of neuroprotection elicited by the blockage of NR2B-NMDAR [103,104]. It is, therefore, reasonable to suspect that, at least in some types of maturing neurons, changes in Ca^2+^ signaling elicited by ketamine occur through the drug interaction with NR2B-containing NMDA receptors.

#### 3.3.2. Effects Facilitated by AMPA Receptors

Several studies further delineated the possible signaling pathways that mediate calcium- and ketamine-dependent alterations in neurogenesis. In early development stages, the number of silent shaft synapses containing NMDARs but not AMPA receptors, was found to be increased [105]. The low-level stimulation of these NMDA receptors is thought to inhibit AMPA receptor trafficking, but strong activation normally overcomes this inhibition during development. The knockdown of NMDAR or their pharmacological blockage disinhibited AMPA receptor trafficking and increased the number of functional synapses enriched in these receptors [106]. Ketamine increased the expression of GluA1 subunit of Ca^2+^-permeable AMPA receptor in the hippocampus and in human dopaminergic neurons differentiated from induced pluripotent stem cells [107,108]. These studies, together with the most recent one [109], point out that AMPA receptors may be another synaptic target for ketamine, and support the hypothesis that their activation is required for antidepressant effect of ketamine, which is largely mediated by BDNF. In particular, ketamine failed to exert its effects in mice with *Bdnf* gene knockdown and when BDNF-neutralizing antibodies were infused into prefrontal cortex, both suggesting that BDNF release was involved in the actions of ketamine [3]. Indeed, ketamine-stimulated BDNF secretion was accompanied by increased CA1 pyramidal cell dendritic spine size and the formation of new dendritic spines and synapses in the prefrontal cortex within 24 h [3,110]. In primary neuronal cultures, activation of AMPA receptors was prerequisite for BDNF release in Ca^2+^-dependent manner, but this effect also required the engagement of L-type VDCCs [111]. In line with that, blockage of Ca^2+^ influx through these channels by verapamil completely inhibited the release of BDNF caused by ketamine. Inhibition of these channels also impaired proliferation of hippocampal neural progenitor cells, as they are thought to sense excitatory neural activity thereby providing a coupling between Ca^2+^ signal, excitation and neurogenesis [112].

Ca^2+^ influx through ketamine-targeted voltage-dependent calcium channels has also been shown to regulate the transcription of BDNF mRNA. Thorough research disclosed that this regulation involved Ca^2+^-dependent binding of cAMP response element binding protein (CREB) and calcium response factor (CaRF) to the BDNF promoters [113,114]. Further study demonstrated the ketamine decreased CREB phosphorylation in the hippocampus [115] which, together with altered BDNF-TrkB receptor-CREB signaling axis, is thought to be associated with long-term memory impairments upon prolonged ketamine expose in neonates [116].

## 4. Ketamine and Synaptic Transmission

An interesting aspect of ketamine action is the effect exerted on Ca^2+^-dependent synaptic transmission, although elucidation of signaling processes creates some difficulties associated with application regimen and drug doses [8]. Therefore, any effects on neuronal transmission should be analyzed taking into account ketamine’s life-time, which is around 2–4 h, as well as the time necessary for complete elimination not only ketamine, but also its metabolites, which was estimated to last from 24 h up to a few days [5]. 

The effects of ketamine on CNS functioning have been widely documented and can be divided into two steps: Observed after short time and observed over longer time period. The latter includes modifications in synaptic plasticity and changed neuronal response that, in addition, appears to be age- and brain region-specific [117,118]. The available data clearly showed that ketamine can alter both, synaptogenesis and synapse function. The first is linked with changed expression of genes involved into formation of proper synapse structure and composition, whereas the second can locally fine-tune the synaptic communication [119,120,121]. Due to the individual profile of receptors and neurotransmitters in different types of neurons, the final response can vary significantly [49,122]. It should be noted that in every case, Ca^2+^ participates directly or indirectly in synaptic signaling processes. An excessive release of glutamate induced by Ca^2+^ dysregulation within the presynaptic compartment causes a prolonged activation of postsynaptic AMPA receptors and some long-term action of ketamine induced by altered ion homeostasis can modify synaptic transmission, due to altered expression of genes coding components of individual signaling pathways [123]. An additional important factor is the effective concentration of ketamine and the frequency of dosing [124].

It has been widely reported that, besides NMDA receptors, ketamine possesses multiple targets with more or less unique action [125]. They include elements which are known to be directly (L-type VDCCs) or indirectly (AMPA receptor, sigma-1 receptor, nicotinic acetylcholine receptor, cyclic nucleotide gated channels and many others) regulated by Ca^2+^. Among them, mTOR (mammalian target of rapamycin) pathway and calcium/calmodulin—Dependent protein kinase II are of particular interest for synaptic transmission and neuronal plasticity.

### 4.1. Ketamine and mTOR Signaling

**Figure 3:**
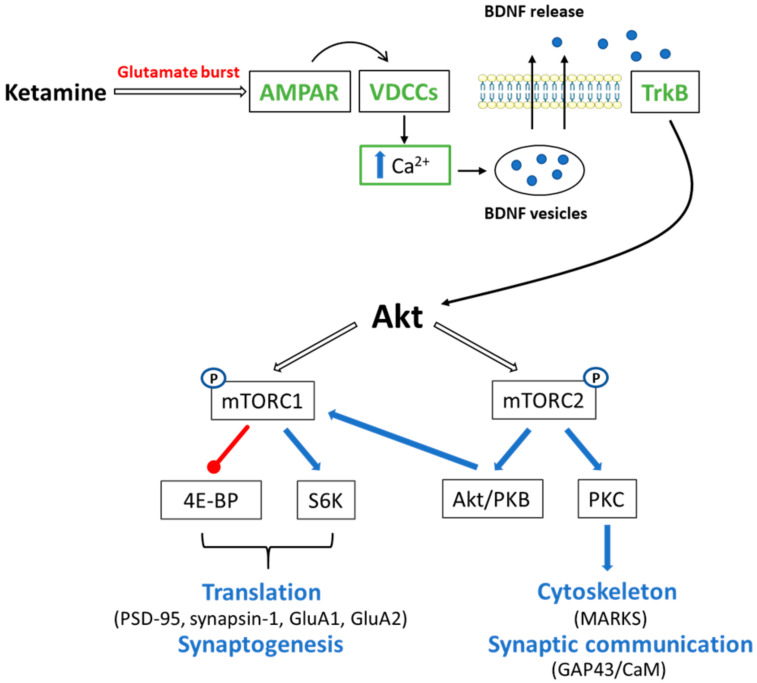
Schematic action of ketamine on mTOR pathway. Ketamine blockade of NMDARs on tonic firing GABA interneurons results in disinhibition of glutamate transmission causing a subsequent glutamate burst. This rapid glutamate burst activates AMPARs and leads to subsequent Ca^2+^ influx through L-type voltage-dependent Ca^2+^ channels (VDCCs). Ca^2+^ promotes BDNF release, which acts via Tropomyosin receptor kinase B/Protein kinase B (TrkB-Akt) to stimulate mTOR signalling. The different effects are linked with mTORC1 and mTORC2 activity. mTORC1, by phosphorylation, activates the ribosomal protein S6 kinase (S6K), as well as represses the inhibitory 4E binding proteins (4E-PB). This concert of events increases the translation of synaptic proteins (PSD95, synapsin-1, GluA1, GluA2) promoting synaptogenesis and intensifying translation of BDNF. Akt/PKB and PKC isoforms are the main substrates regulated by mTORC2. PKC-dependent phosphorylation of the growth-associated protein 43 (GAP43) and MARKS plays an important role in rearrangement of actin cytoskeleton and synaptic transmission. Close relation between GAP43 and calcium-binding protein - calmodulin (CaM) also affects several Ca^2+^/CaM-regulated neuronal processes. Additionally, AKT/PKB can phosphorylate the mTORC1 augmenting it activation.

One of the functionally important target for ketamine is mTOR pathway that regulates normal neuronal growth by promoting differentiation processes, neurite development and synapse formation [126]. mTOR is a serine/threonine kinase and exists in two distinct multiprotein complexes: mTORC1 and mTORC2, which differentially modulate postsynaptic events and presynaptic release of neurotransmitters to control glutamatergic synaptic transmission [127,128]. From a functional point of view, the critical and irreplaceable component of mTORC1 is Raptor (regulatory associated protein of mTOR), while for mTORC2 activity, the most important role is played by Rictor (rapamycin-insensitive companion of mTOR). The mTOR pathway is triggered following the activation of AMPA and/or neurotrophic tyrosine factor receptors (TrkB), thereby initiating the transmission of signals to multiple intracellular systems regulating neuronal processes [129]. Ketamine has been shown to enhance BDNF and mTOR protein levels in brain, thus modifying synaptic transmission [128]. While, mTORC1 and mTORC2 activation exerts different effects on post- and presynaptic functions, which depend on what downstream targets of each complex mediate these changes (Figure 3). The evidence showed that ketamine may improve synaptogenesis through mTOR-stimulated translation of proteins required for neuronal plasticity.

#### 4.1.1. mTORC1

The mTORC1 complex is localized in dendrites and cell bodies, and plays a significant role in promoting activity-dependent mRNA translation near synapses. In the plethora of neuronal processes, mTORC1 functions to regulate protein synthesis and cell growth through downstream molecules: p70S6 kinase (S6K) and elongation factor 4E binding protein (4E-BP) [130]. A low dose of ketamine (10 mg/kg) rapidly activated mTOR signaling and this activation included increased levels of phosphorylated and activated forms of 4E-BP1, p70S6K and mTOR itself. However, the level of phosphorylation was only transient, returning to the pre-stimulatory level after two hours of ketamine administration. Subsequently, increased synaptic protein expression and synapse number at dendritic spines in the prefrontal cortex were observed. This effect was eliminated by pre-treatment with rapamycin, confirming the involvement of mTOR in ketamine action [101,131]. 

mTORC1 regulates synthesis of AMPA receptors (GluA1 and GluA2) increasing their cell surface expression and synaptic activity [132]. Inhibition of NMDA receptors by ketamine and subsequent activation of AMPA-type glutamate receptors induced calcium influx by voltage-dependent Ca^2+^ channels (VDCCs) [111]. Increased Ca^2+^ is a known activator of PI3K/Akt pathway, thereby stimulates the synthesis and release of BDNF, which in turn, act on mTORC1. Interestingly, BDNF is secreted pre-synaptically and post-synaptically in Ca^2+^-dependent manner [133]. The effects of ketamine action are opposite to the synaptic deficits observed under stress conditions and could be responsible for the fast antidepressant effects observed in the prefrontal cortex of rats [131,134]. 

It has been reported that ketamine affected the level of presynaptic protein - synapsin I, as well as postsynaptic protein PSD95 [101]. The presynaptic actions of BDNF involve synapsin I, a protein that associates with synaptic vesicles and regulates the release of various neurotransmitters [135]. Two potential mechanisms have been proposed for BDNF-induced increase in transmitter release, i.e., phosphorylation of synapsin I and TrkB-initiated PLC*γ* activation, leading to Ca^2+^ mobilization from IP_3_-sensitive stores in the ER [136,137]. 

Postsynaptic density protein 95 (PSD-95) belonging to the membrane-associated guanylate kinase (MAGUK) family, is mainly localized in the spines and regulates the dendritic spine size and shape at synapses [138]. For that reason, PSD95 is used as a marker for dendrite branching and remodeling. At a molecular level, PSD-95 is responsible for the trafficking and localization of AMPA and NMDA receptors expressed at the postsynaptic membrane [139]. For example, PSD-95 mediates postsynaptic localization of ~40% of the AMPARs in pyramidal cells of the hippocampal CA1 region [140]. In addition, as a scaffolding protein, through modulation of AMPA receptors may also regulate synaptic plasticity, including long-term potentiation and long-term depression processes, which play a central role in learning and memory. 

#### 4.1.2. mTORC2

Less data are available regarding ketamine action on mTOR complex 2. mTORC2 has been shown to regulate the cell survival and play a role in the maintenance of the actin cytoskeleton, thereby can control the morphological state of dendritic spines [141].

Among presynaptic effects of mTORC2, regulation of Akt/PKB and PKC isoforms activity appears to be the most known. The experiment using mutated mice with a Purkinje cell–specific elimination of *rictor* showed significant changes in synaptic function, a phenomenon accompanied by the loss of activation of Akt and some conventional Ca^2+^-stimulated PKC isoforms [142]. The enzyme plays a critical role in several forms of synaptic plasticity [143]. Enhanced phosphorylation of PKC by mTORC2 was reported to be associated with LTP [144].

Two important PKC substrates—GAP43 (growth-associated protein 43) and MARCKS (myristoylated alanine-rich C-kinase substrate)—have been revealed to be engaged in ketamine-induced effects. Both proteins can interact with actin filaments through electrostatic interactions of their basic domain, thereby can bind and regulate the accessibility of acidic phospholipids, including PIP_2_, a precursor of second messengers: inositol triphosphate (IP_3_) and diacylglycerol (DAG), which participate in the regulation of neuronal Ca^2+^ homeostasis [145].

GAP43 (neuromodulin) is synthesized in the neuronal cell body and becomes highly enriched in the tips of extending neurites and is a protein implicated in axonal growth. Interestingly, its expression increased with ketamine treatment. GAP43 shows a strong affinity for calmodulin in low Ca^2+^ concentration, but calmodulin (CaM) is released when cytosolic Ca^2+^ increases [146]. Under resting conditions GAP43 is bound to CaM, the most ubiquitous and conservative among Ca^2+^ -binding proteins. Increased Ca^2+^ concentration and phosphorylation of GAP43 by PKC leads to the release of CaM from this complex, which in turn, can trigger multiple Ca^2+^/CaM-regulated downstream processes [147,148]. It is plausible that altered GAP43/pGAP43 ratio is the upstream switch for the number of downstream events in neuronal signaling. It also suggests that GAP43, the abundant CaM reservoir, could be indirectly controlled by mTORC2.

The MARCKS belongs to the most known primary targets for protein kinase C which is enriched both within axon terminals and dendritic spines. It plays an essential role in the regulation of actin cytoskeleton, neurite outgrowth, endo- and exocytosis [149]. Upon phosphorylation by PKC or Ca^2+^-dependent binding of calmodulin, MARCKS can activate various signal transduction pathways [150]. In mature neurons, MARCKS has been shown to concentrate PIP_2_ in the post-synaptic compartment. This strongly suggests that MARCKS functions to modulate postsynaptic signaling cascades. In addition, interaction of MARCKS with synapsin I, observed in presynaptic terminals, indicates that MARCKS can act pre- as well as postsynaptically [150].

### 4.2. Ketamine and Ca^2+^/CaM Signaling in Memory Processes

Nowadays, ketamine is used clinically, not only because of its anesthetic properties, but also for the treatment of depression, seizures, chronic pain or bipolar disorder. As mentioned above, the functioning of several neuronal proteins can be modified by ketamine to improve or attenuate the improper interplay between synapse dynamics and function. Since the disturbances in signaling transmission are specifically linked to the type of disease, ketamine-dependent actions could involve diverge mechanisms to re-establish a formation of neural circuits and information storage in the nervous system. A particular role is played by LTP and LTD processes, being the cellular basis for learning and memory. Although, they are active in distinct brain regions, the most operative are in hippocampus and cerebellum.

Principal mediators of LTP are NMDA-type glutamate receptors with subsequent Ca^2+^ influx, which activates CaMKII by phosphorylation at the Thr286 [151]. In turn, CaMKII phosphorylates the NMDA receptors increasing Ca^2+^ entry, providing control of several canonical Ca^2+^-regulated cascades, i.e., PKA, PKC, PI3K or mTOR. It has been demonstrated that AMPA receptors are also necessary for both, LTP and LTD. The up-regulation of AMPARs function could be due to their increased insertion to the synapses and/or by modification (i.e., phosphorylation) of existing synaptic receptors [152]. AMPARs are made up of four subunits, of which GluA1 and GluA2 appear to be a decisive in functional plasticity. Although, AMPARs composition vary among different brain regions, most of subtypes are phosphorylated by PKA, PKC and CaMKII. The prominent effect of LTP and LTD is GluA1 phosphorylation at Ser 831 and Ser 845. Phosphorylation of GluA1 at S831 by CaMKII (and PKC) has been shown to increase AMPAR conductivity during LTP [153]. LTP-induced changes in spine morphology have been reported to be regulated by BDNF-TrkB pathway [154]. Increase in glutamatergic synapse activity stimulates the release of BDNF, which controls the assembly of TrkB-PSD95 complex and up-regulates translation process. It should be underlined that PSD-95 is recognized as the major scaffolding protein for NMDA and AMPA receptors at glutamatergic synapses [17].

LTP-induced dendritic synthesis, translocation and insertion of the AMPARs into postsynaptic membranes modify the morphology and density of postsynaptic spines, but stabilization of a new structure depends on AMPARs attachment to postsynaptic density proteins. There is compelling evidence that binding of the AMPAR to PSD-95 provides required stabilization and functionality, and appropriate levels of PSD-95 are obligatory for activity-dependent synapse stabilization after initial phases of synaptic potentiation [17].

It is well-documented that depression is caused by complex pathological processes in certain brain regions (hippocampus, prefrontal cortex, amygdale), triggered by dysregulation of a variety of signaling pathways (many of them yet unidentified), and characterized by decreased neuronal branching and plasticity in the hippocampus [155]. Prolonged stress conditions can impair LTP and enhance LTD in rat hippocampus [156]. A number of reports have shown that ketamine can alter the activity of several important components of synaptic transmission that contribute to LTP and LTD induction or maintenance under depression-induced memory dysfunction. It was demonstrated that ketamine treatment significantly increased AMPA/NMDA receptor density ratio in the hippocampus of depression-like rats thereby can reverse disease-induced changes [157].

An interesting mechanism of noticeable antidepressant CaMKII function has been recently proposed by Adaikkan et al., [158]. They showed that the administration of ketamine (5 mg/kg) led to differential regulation of CaMKII function, manifested as autoinhibition (T305 phosphorylation) followed by autoactivation (T286) of CaMKII in the hippocampus and cortex. Moreover, in the hippocampus ketamine antidepressant activity can induce rapid and persistent enhancement of neuronal plasticity by deactivation of eukaryotic elongation factor 2 (eEF2) by Ca^2+^/CaM-dependent eEF2 kinase what resulted in enhanced global protein synthesis. The inhibition phase of CaMKII, which lasted 10 to 20 min after the administration of ketamine, occurred concurrently with eEF2K-dependent increased protein synthesis. 

Using an LPS (lipopolysaccharide)-induced model of depression, it was found that ketamine antidepressant effects were associated with decreased extrasynaptic CaMKIIα activity, as well as with altered extrasynaptic GluN2B localization and phosphorylation. In addition, ketamine action delayed induction of GluA1 by 24 h, which was regulated by the activation of CaMKII. Ketamine administration upregulated the expression of p-CREB and BDNF in the hippocampus, preventing the impairment of LTP induction and loss of the synaptic proteins induced by LPS [159]. The above data confirm that Ca^2+^ and calmodulin effectively contribute to the control and regulation of synaptic processes. Ketamine is undoubtedly an interesting alternative in the treatment of depression as it has a mechanism of action different from standard antidepressants. The drug, as an antidepressant, has attracted much attention and showed remarkable results in clinical practice as reviewed in [160]. However, despite low doses of ketamine used in depression studies, a special caution should be taken when used repeatedly. This is because long-term use of ketamine has been linked with urological toxicity, cognitive deficits, hepatic dysfunction and dependency risk [161]. The adverse effects may also include the emergence reactions such as out-of-body experience, hallucinations or dreams, usually seen by recreational users. This concerns are also reflected in the scientific literature about chronic pain, in which investigators conclude that ketamine use should be restricted because of side-effects [162].

## 5. Concluding Remarks

Proper control of [Ca^2+^]_i_ affecting downstream Ca^2+^ signaling is largely responsible for a number of neuronal functions. Therefore, it is not surprising that even mild deficits in Ca^2+^ handling machinery may lead to significant structural and functional deficits in neurons. In some cases, these deficits are temporary and reversible, but in others they may become permanent and trigger irreversible neurodegenerative processes. As reviewed in this article, in vitro and in vivo research using different approach and experimental animal models has indicated that imbalanced Ca^2+^ homeostasis is tightly associated with ketamine action in the brain. Moreover, a growing body of evidence indicates that Ca^2+^ is either directly or indirectly engaged in ketamine-related processes, mostly in NMDA and AMPA receptor-driven neurotransmission. This seems apparent as ketamine is a well-known NMDAR blocker. However, several prolonged effects seem to arise from other “post-drug” effects, not necessarily limited to NMDA receptors but involving Ca^2+^-related, activity-induced structural rearrangements in synaptic connectivity and function. The knowledge of impairments in neuronal Ca^2+^ upon ketamine administration can sustain the needs for its careful clinical use and validation of animal models of ketamine-induced diseases.

## Figures and Tables

**Table 1:** Regional changes in gene expression in rat brain elicited by chronic ketamine administration at 30 mg/kg. The data have been extracted from [28].

	Genes Upregulated	Genes Downregulated
cortex	Cacna1c, Orai1, PMCA4, SPCA1, MCU	PMCA2, SERCA3, NCX3
cerebellum	Cacna1d, Cacna1h, PMCA1, SERCA3, NCX1	Cacna1b, STIM1, PMCA3, MCU
hippocampus	Cacna1d, SERCA3, NCX3	TRPC1, Cacna1b, PMCA3, MCU
striatum	TRPC1, PMCA1, SERCA3, MCU	TRPC6, Cacna1b, Cacna1c, Cacna1d, STIM1, PMCA3, PMCA4, SPCA2, NCX2

Cacna1b—N-type VDCC subunit α-1B; Cacna1c—L-type VDCC subunit α-1C; Cacna1d—L-type VDCC subunit α-1D; Cacna1h—T-type VDCC subunit α-1H; PMCA—Plasma Membrane Ca^2+^-ATPase; SPCA—Secretory Pathway Ca^2+^-ATPase; SERCA—Sarco/endo plasmic reticulum Ca^2+^-ATPase; STIM—stromal interaction molecule; ORAI—calcium release-activated calcium channel; MCU—mitochondrial calcium uniporter; NCX—Na^+^/Ca^2+^ exchanger; TRPC—transient receptor potential canonical channel.
